# Diaphragm excursions as proxy for tidal volume during spontaneous breathing in invasively ventilated ICU patients

**DOI:** 10.1186/s40635-023-00553-z

**Published:** 2023-10-27

**Authors:** Matthijs L. Janssen, Annemijn H. Jonkman, Myrte Wennen, Evert-Jan Wils, Henrik Endeman, Leo Heunks

**Affiliations:** 1 Department of Intensive Care, Erasmus Medical Center, Rotterdam, The Netherlands; 2 Department of Intensive Care, Franciscus Gasthuis & Vlietland, Rotterdam, The Netherlands

**Keywords:** Respiratory failure, Diaphragm excursion, Tidal volume, P-SILI, Non-invasive respiratory support, High-flow nasal oxygen

## Abstract

There is a need to monitor tidal volume in critically ill patients with acute respiratory failure, given its relation with adverse clinical outcome. However, quantification of tidal volume in non-intubated patients is challenging. In this proof-of-concept study, we evaluated whether ultrasound measurements of diaphragm excursion could be a valid surrogate for tidal volume in patients with respiratory failure. Diaphragm excursions and tidal volumes were simultaneously measured in invasively ventilated patients (*N* = 21) and healthy volunteers (*N* = 20). Linear mixed models were used to estimate the ratio between tidal volume and diaphragm excursion. The tidal volume–diaphragm excursion ratio was 201 mL/cm in ICU patients [95% confidence interval (CI) 161–240 mL/cm], and 361 (294–428) mL/cm in healthy volunteers. An excellent association was shown *within* participants (*R*^2^ = 0.96 in ICU patients, *R*^2^ = 0.90 in healthy volunteers). However, the differences between observed tidal volume and tidal volume as predicted by the linear mixed models were considerable: the 95% limits of agreement in Bland–Altman plots were ± 91 mL in ICU patients and ± 396 mL in healthy volunteers. Likewise, the variability in tidal volume estimation *between* participants was large. This study shows that diaphragm excursions measured with ultrasound correlate with tidal volume, yet quantification of absolute tidal volume from diaphragm excursion is unreliable.

## Background

High respiratory effort and tidal volume (TV) have been linked to aggravation of lung injury in patients with acute respiratory failure, also referred to as patient self-inflicted lung injury (P-SILI) [1, 2]. Timely identification of patients with respiratory deterioration may be of clinical relevance [3, 4]. The ROX-index (SpO_2_/FiO_2_/respiratory rate) has been validated to identify patients on high-flow nasal oxygen at risk for requiring endotracheal intubation [5]. Yet, upon increased respiratory loading, changes in TV precede increases in respiratory rate [6]. Indeed, replacing respiratory rate by TV in the ROX-index significantly improved predicting requirement of invasive mechanical ventilation in patients with respiratory failure [7]. However, TV measurement in non-intubated critically ill patients is challenging given the need for accurate airflow measurements.

Ultrasound assessment of diaphragm excursion is reproducible and fair correlations with TV were reported in non-clinical studies [8–10]. Therefore, we hypothesized that bedside measurement of diaphragm excursion could be a valid surrogate for TV. Studies evaluating diaphragm motion have been performed earlier in the context of weaning from mechanical ventilation [11]. The aim of the current proof-of-concept study was to determine the relationship between TV and diaphragm excursions in ICU patients and healthy volunteers. In addition, we investigated correlations between changes in TV and diaphragm excursion within patients.

## Methods

In this prospective study two groups were studied: healthy volunteers and patients on invasive mechanical ventilation, enrolled between August and December 2022. The ethics board approved the study (MEC-2022-0451) and written informed consent was obtained, through legal representatives whenever necessary. Patients with tracheostomy, Body Mass Index (BMI) > 35 kg/m^2^, exacerbation of obstructive lung disease, large pleural effusions (> 1.5 cm), neuromuscular disease or diaphragm paralysis (defined as known paralysis in medical history or having paradoxal diaphragm movement on ultrasound) were excluded.

Simultaneous measurements of TV and diaphragm excursion were obtained in at least 3 breaths per participant. The right hemi-diaphragm was visualized in semi-recumbent position (30 degrees) using subcostal view in M-mode (Sparq, Philips; 2–4 MHz probe) by a single observer experienced in diaphragm ultrasound, as previously described[12]. Images were stored for offline analysis (Sante DICOM Viewer). In patients on invasive mechanical ventilation (Servo-U, Getinge, Sweden) measurements were performed during the first few minutes of a spontaneous breathing trial with positive end-expiratory pressure (PEEP) of 5 cmH_2_O and no inspiratory pressure support. Healthy volunteers were breathing through a mouthpiece with flow sensor connected to a signal acquisition system (BIOPAC Systems, USA), while wearing a nose clip to prevent air leakage. They were instructed to perform tidal breathing as well as deep breathing at non-maximal levels to generate a range of TV. To maximize precision of the measurements, both TV and diaphragm excursions were determined offline and thus were not read from the ventilator or ultrasound machine directly during the imaging procedure. Diaphragm excursions were determined by measurement of the amplitude of the M-line, while being blinded for the corresponding TV values. TV were extracted from the integral of the inspiratory flow-time curve as exported from the ventilator monitor in ICU patients or as measured with a dedicated transducer in healthy subjects.

Using intra-class correlation coefficient (ICC) analysis, single observer test–retest reliability was assessed for diaphragm excursion in a random sample of 3 healthy volunteers and 3 patients (*n* = 74 breaths) using a two-way mixed model with single measures of agreement. Furthermore, we assessed the stability of the ratio between diaphragm excursion and TV within a subject as a surrogate of measurement reliability, considering that this ratio should not change within the short time interval. To this end, we used 3–5 breaths for all subjects and employed a two-way mixed model with single measures of consistency.

Statistical analysis was performed with R (RStudio, version 4.2.2). A linear mixed model with a random intercept per participant and fixed effect of diaphragm excursion was used to estimate the TV-diaphragm excursion ratio, thereby taking multiple and variable measurements per subject into account. The agreement between the observed TV and the TV as predicted by the linear mixed model was evaluated using a Bland–Altman plot. In addition, to test if the relationship between TV and diaphragm excursion was affected by any participant characteristics, such as age, BMI and chest circumference, these characteristics were individually added to the linear mixed model as fixed effects. For all analyses, a *p* value < 0.05 was considered statistically significant.

## Results

**Fig. 1:**
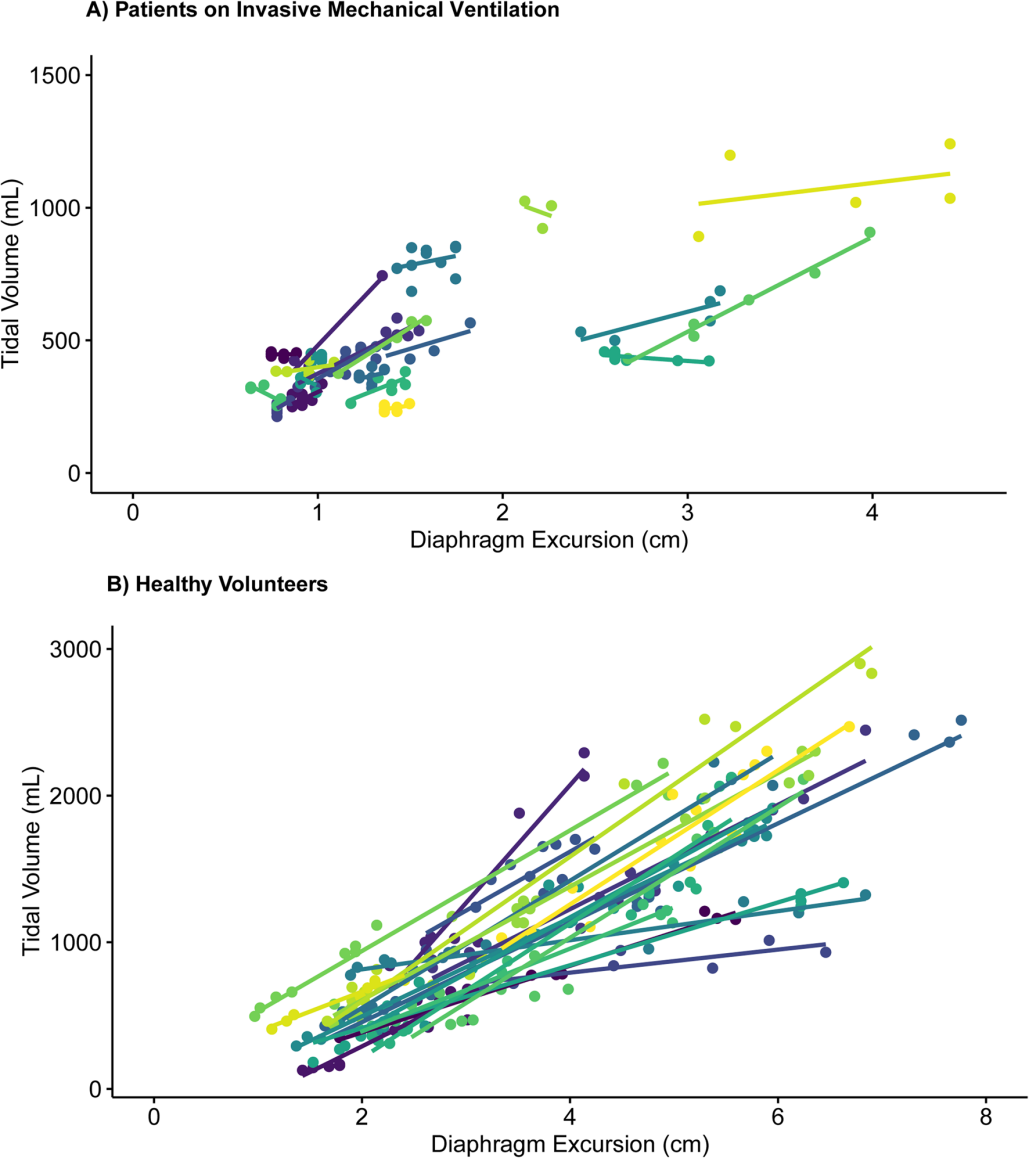
Correlation between Diaphragm Excursion and Tidal volume per participant, separated for ICU patients on invasive mechanical ventilation and healthy volunteers. Every color represents a different participant. **A** Patients on Invasive Mechanical Ventilation. **B** Healthy volunteers

**Fig. 2:**
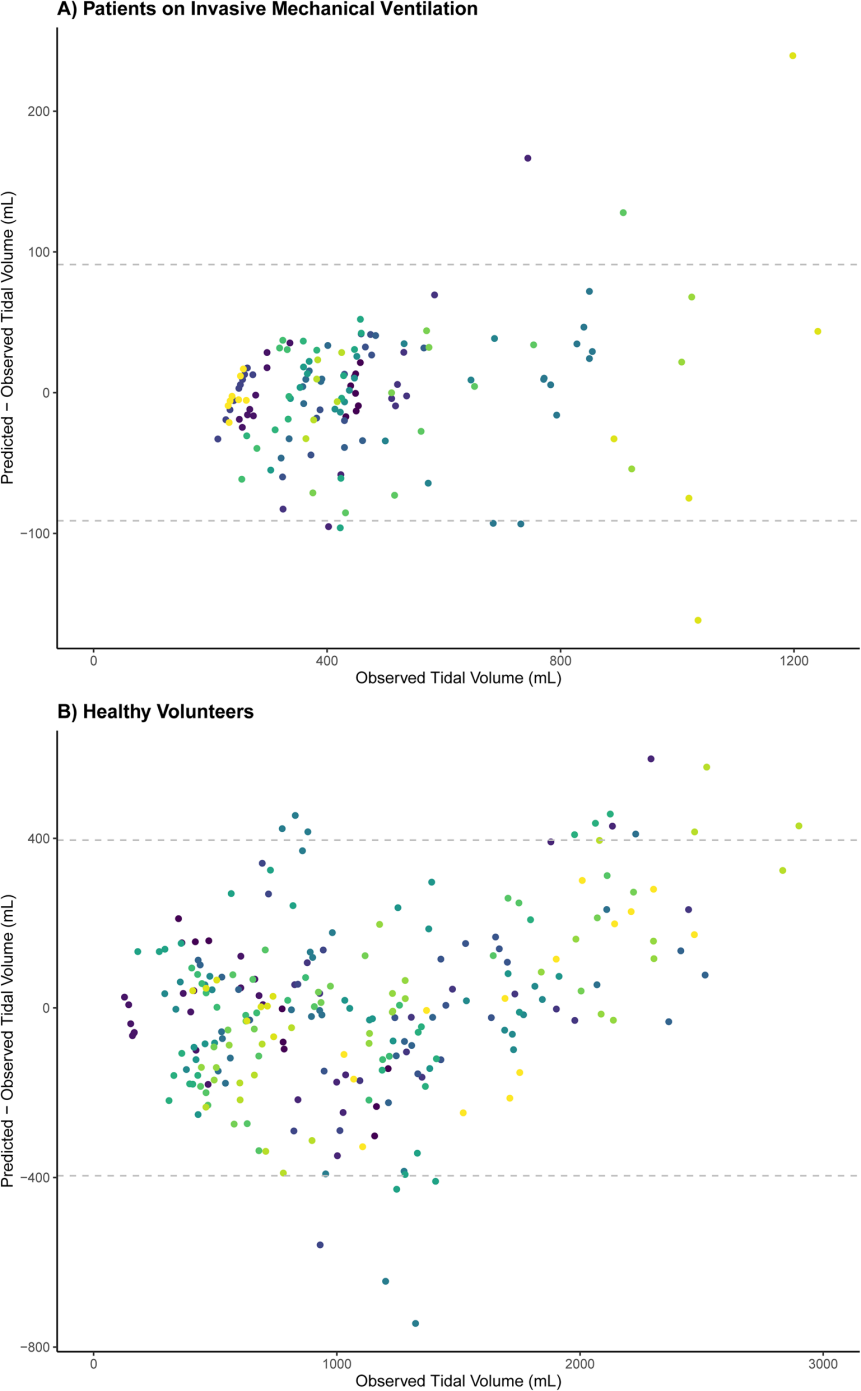
Bland–Altman plot showing the association between observed and predicted Tidal Volumes based on the linear mixed model, separated for ICU patients on invasive mechanical ventilation and healthy volunteers. Every color represents a different participant. The dashed lines indicate 95% limits of agreement: ± 91 mL in ICU patients (**A**) and ± 396 mL in healthy volunteers (**B**)

ICU patients (*N* = 21) and healthy volunteers (N = 20) (Table 1) yielded 139 (median 6, IQR 5–8) and 255 (median 13, IQR 11–14) analyzable breaths, respectively. A good stability of the TV-diaphragm excursion ratio over consecutive breaths within one subject was shown (ICC: 0.86). The mean (± standard deviation) difference between first and second diaphragm excursion measurement was 0.035 ± 0.20 cm. The ICC for intra-observer variability between the first and second measurement of diaphragm excursion was 0.99. The models indicated an excellent association *within* participants (*R*^2^ = 0.96 in invasively ventilated patients, *R*^2^ = 0.90 in healthy volunteers), as illustrated in Fig. 1. The TV-diaphragm excursion ratio was 201 mL/cm in ICU patients (95% confidence interval (CI) 161–240 mL/cm), *p* < 0.001, and 361 (95% CI 294–428) mL/cm, *p* < 0.001 in healthy volunteers. The mean (± standard deviation) value of the per-patient intercept of the model was 0 ± 157 mL in ICU patients, and 0 ± 267 mL for healthy volunteers. The variability in TV estimation *between* participants was large: e.g., a diaphragm excursion of 1.5 cm could correspond to a TV between 250 and 750 mL in ICU patients (Fig. 1). Participant characteristics (age, sex, height, Ideal Body Weight, BMI, chest circumference and days on invasive mechanical ventilation) did not affect the relationship between diaphragm excursion and TV (Table 1). Bland–Altman plots (Fig. 2) showed considerable differences between observed and predicted TV (95% limits of agreement: ± 91 mL in ICU patients and ± 396 mL in healthy volunteers).

**Table 1:** Baseline characteristics for all participants, separated for healthy volunteers and ICU patients

	Overall (*N* = 41)	ICU patients (*N* = 21)	Healthy volunteers (*N* = 20)	*p* value for variable in model
Male sex	26 (63)	16 (76)	10 (50)	0.23
Age (y)	33 (28–63)	60 (49–74)	28.5 (26–31)	0.36
Height (m)	1.8 (1.7–1.8)	1.8 (1.7–1.8)	1.8 (1.7–1.8)	0.96
IBW (kg)	71 (62–72)	68 (62–71)	71 (65–75)	0.75
BMI (kg/m^2^)	24 (22–26)	25 (23–28)	23 (22–25)	0.97
Thorax circumference (cm)	94 (85–103)	101 (95–114)	87 (80–91)	0.38
Days on IMV at measurement	NA	3 (1–5)	NA	0.63
pH		7.44 (7.41–7.46)		
PaCO_2_ (mmHg)		39 (38–42)		
PaO_2_ (mmHg)		84 (76–107)		
PaO_2_/FiO_2_		285 (222–372)		
IMV indication				
Securing airway		4 (19)		
Respiratory failure		3 (14)		
Circulatory failure		5 (24)		
Neurological/neurotrauma		7 (33)		
Post-surgery or trauma		2 (10)		

Characteristics were added to the linear mixed model with ICU patients as separate fixed effects; *p* values represent the statistical effect of these covariates. Categorical data are represented as number with percentage between brackets. Continuous data are represented as median with quartiles. *IMV* invasive mechanical ventilation, *ICU* intensive care unit, *IBW* Ideal Body Weight, *BMI* Body Mass Index, NA: not applicable

## Discussion

Our study demonstrates a correlation between ultrasound measurement of diaphragm excursion and TV in healthy volunteers, but the large variability in the data in ICU patients indicates a less obvious association. This precludes a reliable estimation of the absolute value of TV from diaphragm excursion measurement in the clinical setting.

The difference in TV-diaphragm excursion ratio between ICU patients and healthy volunteers may be explained by a smaller distribution of TV in ICU patients. Indeed, an additional (sensitivity) analysis of the TV-diaphragm excursion relationship in healthy volunteers when including only breaths in the same TV range as ICU patients (TV ≤ 1250 mL) indicated that the TV-diaphragm excursion ratio is comparable to ICU patients [231 (209–255) mL/cm]. In addition, altered respiratory mechanics and potential effects of PEEP on diaphragm efficiency [13] have likely played a role. An earlier study showed that the application of PEEP resulted in caudal displacement of the diaphragm and decreased the diaphragm contractile efficiency. Possibly, the decrease in TV-diaphragm excursion ratio in ICU patients compared to healthy volunteers is explained by PEEP.

Our results contrast with earlier studies that observed a fair correlation between TV and diaphragm excursion [9, 10]. However, these studies were performed in non-clinical settings, and used simple linear regression analysis without accounting for multiple measurements per participant. Furthermore, their larger TV-diaphragm excursion ratios (555 and 625 mL/cm, respectively) may be explained by recruitment of accessory muscles, since participants in earlier studies were instructed to inhale up to total lung capacity.

There are limitations of our study to acknowledge. First, we did not quantify accessory muscle use, although the association between diaphragm excursion and TV is affected by these muscles. Occult recruitment of accessory muscles may, therefore, have distorted the TV-diaphragm excursion ratio especially in ICU patients. However, we evaluated the potential of diaphragm excursion as bedside tool to monitor TV. Simultaneous evaluation of accessory muscle recruitment might have improved the understanding of the association between diaphragm excursion and TV, but would also complicate its clinical applicability. Second, the average time on invasive mechanical ventilation in the studied ICU patients was rather short. We recognize that the association between diaphragm excursion and TV may differ in patients with prolonged invasive mechanical ventilation due to diaphragm muscle dysfunction [14]. However, the targeted population to use diaphragm excursion as proxy for TV would concern non-intubated patients rather than those with prolonged invasive mechanical ventilation. Third, we excluded patients with high BMI due to difficulty of imaging the diaphragm, and also patients with exacerbation of obstructive lung disease due to flattening of their diaphragm resulting from pulmonary hyperinflation. This may affect the generalizability of our results as these are common comorbidities in the ICU population. Fourth, images from multiple breaths were obtained once in each participant. The use of a single ultrasonographer may imply that if this method were to be translated to clinical practice more variability from different observers may be introduced. However, the reproducibility of diaphragm excursion measurements via ultrasound has already been substantiated in a large study [8]. Consequently, we reasoned that imaging performed by multiple observers was deemed unnecessary in this study. Finally, TV was derived from the flow tracings but under different gas conditions (body temperature, pressure, water vapor saturated in ventilated patients and ambient temperature and pressure in healthy subjects); this will not affect the primary conclusion and between-subject variability but may result in a slightly higher absolute ratio (mL/cm) for healthy volunteers compared to ventilated patients.

Adequate diaphragm imaging is pivotal to establish the TV-diaphragm excursion ratio in the critical care setting. M-mode ultrasound measures unidimensional diaphragm movement and requires diaphragm motion perfectly aligned with the M-mode line. Even then, commonly employed one-dimensional measures of diaphragm excursion cannot capture the complete diaphragm motion, which is multidimensional. Our study emphasizes the complexity of the resultant relationship between a single measurement of diaphragm excursion and TV. Hence, a one-dimensional measure is unsuitable to determine absolute values or a safe cutoff for TV. Advanced techniques such as speckle tracking may have superior performance by quantifying diaphragm motion in multiple dimensions [15, 16]. Prior studies were often hampered by the application of inspiratory pressure support during ultrasound measurements. However, it should be stressed that such measurements of excursion should be performed in patients without inspiratory ventilator support [12, 17] to reliably reflect the patient’s own contribution to generating TV, such as done in our study. The relationship between diaphragm excursion as measured with speckle tracking and TV and its possible role in predicting the need for intubation in non-intubated patients with acute respiratory failure requires further study.

## Conclusion

To conclude, in this proof-of-concept study in critically ill patients and healthy volunteers, single measurement of diaphragm excursion is not a clinically feasible surrogate for absolute values of TV. Consecutive measurements of diaphragm excursion may indicate changes in TV within patients with respiratory failure, yet its margin of error is too large to use the measurement for monitoring clinical deterioration. Therefore, diaphragm excursions measured with ultrasound should not be used to identify patients at risk for P-SILI.

## Take home message

Monitoring tidal volume in patients with respiratory failure is necessary, but its measurement in non-intubated patients is challenging. This study shows that diaphragm excursions measured with ultrasound correlate with tidal volume, yet quantification of absolute values for tidal volume from diaphragm excursion is unreliable.

## Tweet

Measurements of diaphragm excursions with ultrasound correlate with tidal volume, but should not be used to determine tidal volume.

## Data Availability

The data sets used and/or analysed during the current study are available from the corresponding author on reasonable request.

## Abbreviations

ICU: Intensive care unit
IMV: Invasive mechanical ventilation
P-SILI: Patient self-inflicted lung injury
PEEP: Positive end-expiratory pressure
TV: Tidal volume
BMI: Body Mass Index
IBW: Ideal body weight

## References

[1] Grieco DL, Menga LS, Eleuteri D, Antonelli M (2019). Patient self-inflicted lung injury: implications for acute hypoxemic respiratory failure and ARDS patients on non-invasive support. Minerva Anestesiol.

[2] Yoshida T, Grieco DL, Brochard L, Fujino Y (2020). Patient self-inflicted lung injury and positive end-expiratory pressure for safe spontaneous breathing. Curr Opin Crit Care.

[3] Brochard L, Slutsky A, Pesenti A (2017). Mechanical ventilation to minimize progression of lung injury in acute respiratory failure. Am J Respir Crit Care Med.

[4] Kang BJ, Koh Y, Lim CM, Huh JW, Baek S, Han M (2015). Failure of high-flow nasal cannula therapy may delay intubation and increase mortality. Intensive Care Med.

[5] Roca O, Caralt B, Messika J, Samper M, Sztrymf B, Hernandez G (2019). An index combining respiratory rate and oxygenation to predict outcome of nasal high-flow therapy. Am J Respir Crit Care Med.

[6] Vaporidi K, Akoumianaki E, Telias I, Goligher EC, Brochard L, Georgopoulos D (2020). Respiratory drive in critically ill patients. Pathophysiology and clinical implications. Am J Respir Crit Care Med.

[7] Chen D, Heunks L, Pan C, Xie J, Qiu H, Yang Y (2022). A novel index to predict the failure of high-flow nasal cannula in patients with acute hypoxemic respiratory failure: a pilot study. Am J Respir Crit Care Med.

[8] Boussuges A, Gole Y, Blanc P (2009). Diaphragmatic motion studied by m-mode ultrasonography: methods, reproducibility, and normal values. Chest.

[9] Cohen E, Mier A, Heywood P, Murphy K, Boultbee J, Guz A (1994). Excursion-volume relation of the right hemidiaphragm measured by ultrasonography and respiratory airflow measurements. Thorax.

[10] Houston JG, Angus RM, Cowan MD, McMillan NC, Thomson NC (1994). Ultrasound assessment of normal hemidiaphragmatic movement: relation to inspiratory volume. Thorax.

[11] Parada-Gereda HM, Tibaduiza AL, Rico-Mendoza A, Molano-Franco D, Nieto VH, Arias-Ortiz WA (2023). Effectiveness of diaphragmatic ultrasound as a predictor of successful weaning from mechanical ventilation: a systematic review and meta-analysis. Crit Care (London, England).

[12] Tuinman PR, Jonkman AH, Dres M, Shi ZH, Goligher EC, Goffi A, et al. Respiratory muscle ultrasonography: methodology, basic and advanced principles and clinical applications in ICU and ED patients-a narrative review. Intensive Care Med. 2020;46(4):594–605. 10.1007/s00134-019-05892-8 PMC7103016 31938825

[13] Jansen D, Jonkman AH, Vries HJ, Wennen M, Elshof J, Hoofs MA (2021). Positive end-expiratory pressure affects geometry and function of the human diaphragm. J Appl Physiol (1985).

[14] Vassilakopoulos T, Petrof BJ (2004). Ventilator-induced diaphragmatic dysfunction. Am J Respir Crit Care Med.

[15] Oppersma E, Hatam N, Doorduin J, van der Hoeven JG, Marx G, Goetzenich A (2017). Functional assessment of the diaphragm by speckle tracking ultrasound during inspiratory loading. J Appl Physiol (1985).

[16] Huang D, Song F, Luo B, Wang S, Qin T, Lin Z (2023). Using automatic speckle tracking imaging to measure diaphragm excursion and predict the outcome of mechanical ventilation weaning. Critical Care (London, England).

[17] Sabourin E, Carpentier C, Lai C, Monnet X, Pham T (2023). "Under pressure": should we use diaphragm excursion to predict weaning success in patients receiving pressure support ventilation?. Critical Care (London, England).

[18] World Medical Association Declaration of Helsinki (2013). ethical principles for medical research involving human subjects. JAMA.

